# MS/MS analysis and imaging of lipids across *Drosophila* brain using secondary ion mass spectrometry

**DOI:** 10.1007/s00216-017-0336-4

**Published:** 2017-04-07

**Authors:** Nhu T. N. Phan, Marwa Munem, Andrew G. Ewing, John S. Fletcher

**Affiliations:** 1 Department of Chemistry and Molecular Biology, University of Gothenburg, Kemivägen 10, 41296 Gothenburg, Sweden; 2 Institute for Neuro- and Sensory Physiology, University Medical Center Göttingen, Humboldtallee 23, 37073 Göttingen, Germany; 3 Department of Chemistry and Chemical Engineering, Chalmers University of Technology, Kemivägen 10, 41296 Gothenburg, Sweden

**Keywords:** ToF-SIMS, Tandem mass spectrometry, MS/MS, Lipids, Fly brain, Imaging, IMS/MSI

## Abstract

Lipids are abundant biomolecules performing central roles to maintain proper functioning of cells and biological bodies. Due to their highly complex composition, it is critical to obtain information of lipid structures in order to identify particular lipids which are relevant for a biological process or metabolic pathway under study. Among currently available molecular identification techniques, MS/MS in secondary ion mass spectrometry (SIMS) imaging has been of high interest in the bioanalytical community as it allows visualization of intact molecules in biological samples as well as elucidation of their chemical structures. However, there have been few applications using SIMS and MS/MS owing to instrumental challenges for this capability. We performed MS and MS/MS imaging to study the lipid structures of *Drosophila* brain using the J105 and 40-keV Ar_4000_^+^ gas cluster ion source, with the novelty being the use of MS/MS SIMS analysis of intact lipids in the fly brain. Glycerophospholipids were identified by MS/MS profiling. MS/MS was also used to characterize diglyceride fragment ions and to identify them as triacylglyceride fragments. Moreover, MS/MS imaging offers a unique possibility for detailed elucidation of biomolecular distribution with high accuracy based on the ion images of its fragments. This is particularly useful in the presence of interferences which disturb the interpretation of biomolecular localization.

**Electronic supplementary material:**

The online version of this article (doi:10.1007/s00216-017-0336-4) contains supplementary material, which is available to authorized users.

**Graphical abstract:**
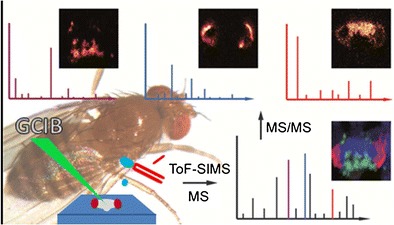
MS/MS was performed during time-of-flight secondary ion mass spectrometry (ToF-SIMS) analysis of *Drosophila melongaster* (fruit fly) to elucidate the structure and origin of different chemical species in the brain including a range of different phospholipid classes (PC, PI, PE) and di- and triacylglycerides (DAG & TAG) species where reference MS/MS spectra provided a potential means of discriminating between the isobaric [M-OH]^+^ ion of DAGs and the [M-RCO]^+^ ion of TAGs.

## Introduction

Lipids are a very important group of biomolecules performing different functions in biological systems. They are small molecules with masses typically up to about 1000 Da. There are more than 1000 lipid species in a eukaryotic cell [1]. Among different lipid groups, glycerophospholipids are the main component of the lipid bilayers of cell membranes besides cholesterol. Over 400 glycerophospholipids with different structures have been identified in a single cell [2]. The highly complex lipid compositions in biological systems play important regulatory roles in different cellular processes, for instance inducing cell membrane fusion by regulating membrane curvature, mediating secondary messengers for cellular signaling, and supplying energy for the biological system. Moreover, it has been shown by numerous studies that lipid perturbation is directly involved in various neurological disorders and diseases such as ischemic stroke, Alzheimer’s disease, attention deficit hyperactivity disorder (ADHD), and schizophrenia [3–8]. It is therefore critical in biological research to obtain information about the molecular structures of specific lipids which are relevant for a biological process or pathway under study.

Secondary ion mass spectrometry (SIMS), one of the variants of imaging mass spectrometry (IMS, also referred to as mass spectrometric imaging, MSI), is an attractive imaging technique for visualizing the distribution of chemical components in samples, from inorganic samples to organic materials including biological tissues and single cells. To perform SIMS imaging, a focused primary ion beam impacts the sample surface which results in the ejection of secondary ions from the sample. The secondary ions are then separated in the mass spectrometer and detected by their specific mass to charge ratio (*m/z*). SIMS offers unique advantages for chemical imaging, especially high chemical specificity, sensitivity, applicability to all kinds of materials, and nanometer to micrometer range spatial resolution depending on the primary ion source used. In biological research, SIMS has been increasingly recognized as a useful approach to study biological processes, biomarkers of diseases, and pharmacology [9–11]. The technique has been rapidly and continuously developing to meet the expectations and needs of various research areas.

Due to the high complexity of biological samples, which commonly results in overlapping peaks between the ion species of interest and interferences, a current demand in biological research is the identification of the imaged biomolecules. In imaging mass spectrometry, this can be performed concurrently or after the imaging experiment. In conventional approaches, similar samples are examined in parallel, one for imaging and the other for MS/MS experiments using a bulk solution analysis mass spectrometer such as ESI-MS or APCI-MS. Ideally, MS/MS experiments can be simultaneously performed on the same sample in the IMS instrument to increase the reliability of the results and the convenience in measurement. For this purpose, MS/MS capability has been one of the goals in instrumental development of IMS. To perform MS/MS, the signals of MS precursors must be sufficiently high to obtain MS/MS spectra. This is especially true for high mass species such as the molecular ions of lipids.

MS/MS has been done in imaging mass spectrometry, especially with MALDI ion sources [12, 13]. However, MS/MS is relatively new in SIMS imaging [14] and there are very few real biological applications to date using SIMS and MS/MS. Two instruments are now available for static SIMS with MS/MS analysis, including a new instrument from PHI (Physical Electronics, USA) and the J105 *3D Chemical Imager* (Ionoptika, UK) [15], where MS/MS has so far been demonstrated on zebra finch brain and zebrafish whole body [14], metabolites [16], and secreted signaling molecules on bacteria [17]. A key factor in using MS/MS for SIMS imaging, especially for lipids where intact molecules are of interest, is the ability to generate sufficient numbers of molecular ions as precursors and this can be accomplished with cluster ion sources.

Among the variety of cluster ion sources, the gas cluster ion beam (GCIB) sources such as Ar_*n*_^+^, (CO_2_)_*n*_^+^ (H_2_O)_*n*_^+^, etc. are the best choice to obtain high sensitivity for MS/MS of intact molecular ions as well as for their MS imaging. GCIB beams (40 keV) have shown improved signal levels for large molecules owing to its remarkably reduced fragmentation of the secondary ions compared to equivalent energy polyatomic ion beams (C_60_^+^) [18–20]. The high-energy 40-keV Ar GCIB has shown an increase in signals for ions at *m/z* > 500 Da relative to the 40-keV C_60_^+^ gun, and a spatial resolution <3 μm was achieved. It has been used on mouse brain [21] and human cancer biopsy samples [22] and to study lipid structural changes in *Drosophila* brain when treated with the stimulant drug methylphenidate [23].

In this paper, we image and identify molecular lipids by the length and saturation level of fatty acid chains, with the ability to annotate the lipid classes based on their headgroup type. Furthermore, GCIB is used to increase the possibility of MS/MS imaging in order to elucidate the spatial distributions of the biomolecules and their MS/MS product ions. We demonstrate one of the first biological applications of ToF-SIMS MS, MS/MS profiling, and imaging, using the J105 and 40-keV Ar_4000_^+^ GCIB. Here, we perform MS and MS/MS imaging to study the lipid structures of *Drosophila* brain, with the novelty being the use of MS/MS SIMS analysis of intact lipids in the fly brain. We have used this to obtain a detailed analysis of the phospholipid structures observed in the fly brain with a focus on diacylglycerides and glycerophospholipids and the use of MS/MS for imaging the brain. A major advantage of this method is shown to be an ability to screen isobaric interferences.

## Experimental

### Fly culture and sample preparation for SIMS imaging

Transgenic *Drosophila* flies (TH-GFP) were cultured on potato meal/agar medium. Detailed fly culturing protocols can be found in the previous literature [23, 24]. After culture, the flies were loaded onto a fly collar (4M Instrument & Tool LLC, USA), which kept all the fly heads in the same orientation. The fly collar was then embedded in 10% gelatin (Sigma-Aldrich, Stockholm, Sweden), which was subsequently solidified and frozen at −80 °C for storage overnight. The frozen gelatin block containing the fly heads was then further cooled in liquid nitrogen, detached from the fly collar, and subsequently sectioned using a cryo-microtome (Leica CM 1520, Leica Biosystems) at −20 °C to produce slices of 15-μm thickness in the dorsal direction. The brain sections were placed onto indium tin oxide-coated microscope slides, allowed to thaw in a vacuum desiccator for about 3 h, and transferred into the J105 SIMS instrument (Ionoptika Ltd., UK) for analysis.

### ToF-SIMS MS and MS/MS measurements

ToF-SIMS imaging and MS/MS measurements were carried out using a *J105* ToF-SIMS instrument, for which operation principles are described in more details elsewhere [15, 25]. A high-energy 40-keV Ar GCIB was used for all experiments in positive and negative modes. The Ar_4000_^+^ cluster contained 8% CO_2_ to improve cluster formation. A beam size of ~6 μm/pixel with a primary ion current 50 pA was used to acquire the ion images of 128 × 128 pixels. The resulting primary ion dose density was approximately 5.6 × 10^13^ ion/cm^2^. The instrument provided mass resolution (*m*/∆*m*) of ~6000 for *m/z* 798.52.

MS/MS experiments were performed after the MS imaging experiments but on the same sample in the same instrument. The secondary ions, after exiting from the buncher, were fragmented by colliding with inert gas (Ar) in a collision cell. Collision energy ranged between approximately 0.5 and 5 keV depending on the position of the secondary ions in the buncher when the bunching voltage was applied. As the fragmentation occurred in a field-free region, both the precursor and product ions continued to travel in the spectrometer at the same velocity and were selected by a timed ion gate after a short time-of-flight region. The ions were subsequently transmitted through the main time-of-flight analyzer where they were separated based on different *m/z* before arriving at the detector (ToF-ToF configuration for MS/MS). The exact collision gas pressure cannot be measured but can be indirectly monitored using a cold cathode gauge that was used for monitoring pressure in the ToF analyzer. For each MS/MS experiment, only one precursor was chosen. The mass resolution in MS/MS was typically in the range 2500–3500 for the MS precursor from *m/z* 500 to 800 Da.

## Results and discussion

Lipids are an important group of pharmaceutical and medical targets owing to their high abundance and widespread involvement in regulating various cellular functions in a biological systems. In our study, the fruit fly *Drosophila* has been used and its brain lipid structures investigated. *Drosophila* has been used as a model system in an enormous number of studies on cellular mechanisms which underlie human development, behaviors, and diseases [26–29]. The lipid structure of *Drosophila* brain has been imaged by ToF-SIMS showing that brain lipids are altered following the administration of the stimulant drug methylphenidate [23]. A further important step in SIMS imaging that is essential for biological experiments is the possible identification of brain lipids as well as elucidation of their product ion distributions in the fly brain.

### MS and MS/MS analysis of molecular glycerophospholipids in the fly brain

**Fig. 1:**
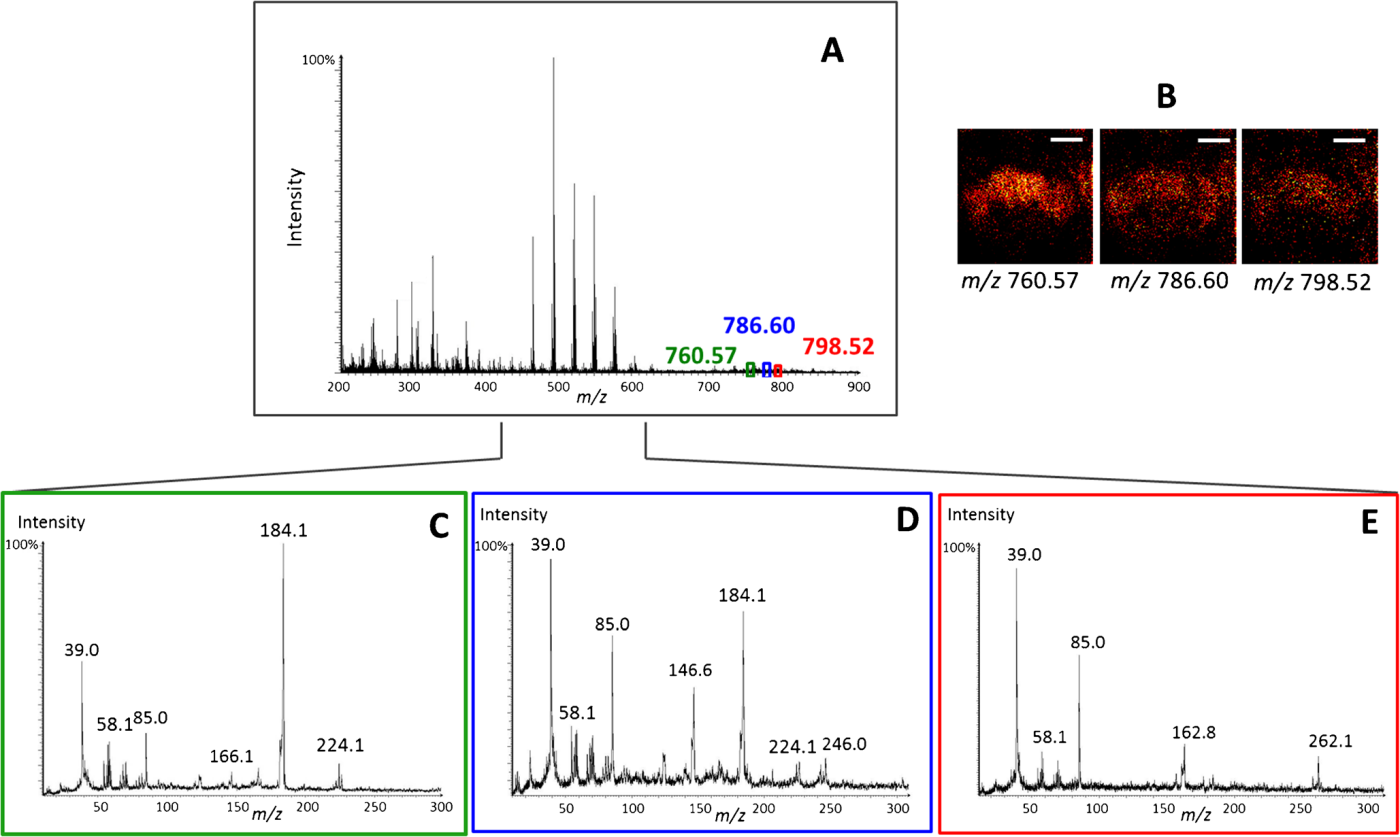
Structural elucidation of phosphatidylcholine lipids in the fly brain from SIMS MS/MS spectra in positive ion mode. (**A**) MS spectrum of a fly brain with selected precursors for MS/MS analysis highlighted at *m/z* 760.57, 786.60, and 798.52. (**B**) SIMS ion images of the selected lipid precursor ions in a fly brain. (**C–E**) SIMS MS/MS spectra of the selected precursors: (**C**) PC(34:1), [C_42_H_83_NO_8_P]^+^, at *m/z* 760.6; (**D**) PC(36:2), [C_44_H_85_NO_8_P]^+^, at *m/z* 786.6; (**E**) [PC(34:1)+K]^+^ at *m/z* 798.5. *Scale bar* is 200 μm. The *m/z* values are quoted to 0.05 for MS and 0.1 for MS/MS. Headgroup fragment ions for PC lipids are [C_3_H_8_N]^+^ at *m*/z 58.1, [C_5_H_15_PNO_4_]^+^ at *m/z* 184.1, and [C_8_H_19_PNO_4_]^+^ at *m/z* 224.1

The advantage of using the 40-keV Ar_4000_^+^ GCIB for these analyses is that high signals are obtained for intact lipid ions. In this section, the MS/MS analyses of the intact *pseudo*-molecular ion lipids of glycerophospholipids including phosphatidylcholines (PCs), phosphatidylethanolamines (PEs), and phosphatidylinositols (PIs) are shown. Glycerophospholipids are the main components of the cell plasma membrane. In brain tissue, glycerophospholipids comprise of 20–25% of the dry weight [30]. The [M+H]^+^ *pseudo*-molecular ions of PC(34:1), [C_42_H_83_NO_8_P]^+^, at *m/z* 760.57, PC(36:2), [C_44_H_85_NO_8_P]^+^, at *m/z* 786.60, and potassium adduct [PC(34:1)+K]^+^, at *m/z* 798.52, were selected from the MS spectra of the fly brain as precursor ions for MS/MS analysis (Fig. 1). PCs distribute across the entire fly brain consistent with the distribution of their headgroup fragment [C_5_H_15_NPO_4_]^+^ at *m/z* 184.07 (Fig. 1B). The main product ions of the precursor PC (34:1) at *m/z* 760.57 and PC(36:2) at *m/z* 786.60 ions are the signature fragments of PC headgroup at *m/z* 184.1 and C_8_H_19_PNO_4_^+^ at *m/z* 224.1, [C_3_H_8_N]^+^ at *m/z* 58.1, and unknown peak at *m/z* 85.0 (Fig. 1C, D). The fragment *m/z* 85.0 could be hydrocarbon breakdown from fatty acid chain. However, as the peak ratio between this fragment and K^+^ is quite consistent (around 1/2 compared to the K^+^ peak) in the MS/MS spectra of the different lipids (Fig. 1C–E), this fragment is most likely a K^+^-containing species. Thus, it is possible to confirm if the molecules are PC lipids by the detected headgroup fragments and to confirm their total carbons of both fatty acid chains as well as their saturation level. From the MS/MS spectra, a significant signal for the K^+^ ion at *m/z* 39.0 is observed. The presence of K^+^ ion is likely from a co-selected species with similar *m*/*z* to the target precursor in MS/MS analysis.

For the salt adduct of lipid [PC(34:1)+K]^+^ at *m/z* 798.52, the main product ion is the K^+^ resulting in a high-intensity K^+^ peak at *m/z* 39.0. Other less significant fragments such as [C_3_H_8_N]^+^ and the fragment at *m/z* 85.0 were also observed (Fig. 1E). From these fragmented peaks, the precursor *m/z* 798.52 is confirmed as the potassium adduct of PC(34:1).

**Fig. 2:**
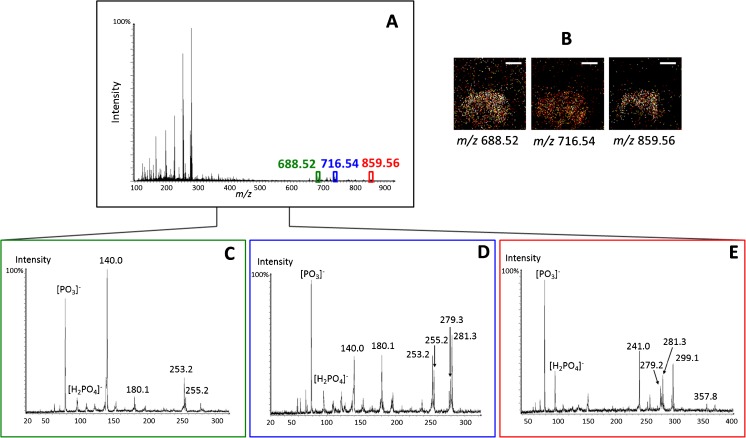
Structural elucidation of phosphatidylethanolamine and phosphatidylinositol lipids in the fly brain from SIMS MS/MS spectra in negative ion mode. (**A**) MS ion spectrum of a fly brain. The lipids of interest for MS/MS are highlighted at *m/z* 688.52, 716.54, and 859.56. (**B**) SIMS ion images of the selected lipids in the brain. (**C–E**) SIMS MS/MS spectra of the selected precursors: (**C**) PE(32:1), [C_37_H_71_NO_8_P]^−^, at *m/z* 688.5; (**D**) PE(34:1), [C_39_H_75_NO_8_P]^−^, at *m/z* 716.5; (**E**) PI(36:3), [C_45_H_80_O_13_P]^−^, at *m/z* 859.6. *Scale bar* is 200 μm. The *m/z* values are quoted to 0.05 for MS and 0.1 for MS/MS. The headgroup fragment ions for PE are [C_2_H_7_NPO_4_]^−^ at *m/z* 140.0 and [C_5_H_11_NPO_4_]^−^ at *m/z* 180.1 and for PI are [C_6_H_10_PO_8_]^−^ at *m/z* 241.0 and [C_9_H_16_PO_9_]^−^ at *m/z* 299.1. Fatty acid fragments from these lipids are [C_16_H_29_O_2_]^−^ at *m/z* 253.2, [C_16_H_31_O_2_]^−^ at *m/z* 255.2, [C_18_H_31_O_2_]^−^ at *m/z* 279.3, and [C_18_H_33_O_2_]^−^ at *m/z* 281.3

In negative ion mode spectra, several PEs and PIs are observed in the fly brain (Fig. 2A). PE(32:1) ([M-H]^−^, [C_37_H_71_NO_8_P]^−^) at *m/z* 688.52 and PE(34:1) ([M-H]^−^, [C_39_H_75_NO_8_P]^−^) at *m/z* 716.54 distribute relatively evenly across the fly brain, whereas PI(36:3) ([M-H]^−^, [C_45_H_80_O_13_P]^−^) at *m/z* 859.56 is more dominant within the central brain and optical lobes (Fig. 2B). The MS/MS analysis shows typical peaks from the headgroups as well as two fatty acid chains of the lipids. Therefore, not only is the type of lipid confirmed but also the number of carbons and the unsaturation level of each fatty acid chain in the lipid structure can be elucidated. For instance, *m/z* 688.52 is assigned as PE(32:1) based on its characteristic fragment ions with the PE headgroup [C_2_H_7_NPO_4_]^−^ at *m/z* 140.0, [C_5_H_11_NPO_4_]^−^ at *m/z* 180.1, fatty acids [C_16_H_29_O_2_]^−^ at *m/z* 253.2, and [C_16_H_31_O_2_]^−^ at *m/z* 255.2 (Fig. 2C). For PE(34:1) at *m/z* 716.54, the MS/MS spectrum shows that the most abundant fatty acids are C16:1, [C_16_H_29_O_2_]^−^ at *m/z* 253.2 and C18:1, [C_18_H_33_O_2_]^−^ at *m/z* 281.3. The additional smaller peaks assigned to fatty acids C16:0, [C_16_H_31_O_2_]^−^, at *m/z* 255.2, and C18:2, [C_18_H_31_O_2_]^−^, at *m/z* 279.3, are probably from PE(34:2) ([M-H]^−^, [C_39_H_73_NO_8_P]^−^) at *m/z* 714.54 which was partially co-selected with the main precursor (Fig. 2D). The structure of PI(36:3) at *m/z* 859.56 was confirmed based on the detection of the PI headgroup ions, [C_6_H_10_PO_8_]^−^ at *m/z* 241.0 and [C_9_H_16_PO_9_]^−^ at *m/z* 299.1, and two constituting fatty acid peaks, C18:2, [C_18_H_31_O_2_]^−^ at *m/z* 279.3 and C18:1, [C_18_H_33_O_2_]^−^ at *m/z* 281.3 (Fig. 2E). In addition, common small fragmented ions representative for phospholipids were also detected in all the MS/MS spectra such as PO_3_^−^ at *m/z* 79.0 and H_2_PO_4_^−^ at *m/z* 97.0.

### MS and MS/MS analysis of possible diacylglycerols (DAGs) in fly brain

Peaks in the *m/*z 450–600 range are commonly observed in the SIMS spectra of biological samples and arbitrarily referred to as DAG peaks. As shown in Fig. 1, these potential DAG peaks are the most intense peaks in the mass spectrum of these fly brain samples. There are two challenges associated with the characterization of these species. The first is the potential range of different combinations of acyl chains that can produce isobaric *m/z* values in the mass spectra. For example, DAG(32:0) may contain two C16 fatty acids or one C14 and one C18 fatty acid. The second challenge lies in the ambiguity in the origin of these species. Although widely referred to as DAGs in the ToF-SIMS literature, the [M-OH]^+^ ion from the DAGs cannot be distinguished from the [M-RCOO]^+^ fragment of triacylglycerols. Reference samples of di- and tripalmitate have been analyzed using MS, and MS/MS of the *m/z* 551 “DAG” peak has been performed in both cases. The MS spectrum of the tripalmitate contains a small peak assigned to the [M+H]^+^ pseudo-molecular ion, while the base peak in the spectrum is at *m/z* 551 and is assigned to the [M-RCOO]^+^ ion. These peaks are consistent with those observed in electron impact spectra in the NIST library and have been known since the 1960s [31]. The dipalmitate produced an intense peak corresponding to the [M-OH]^+^ at *m/z* 551, while the base peak in the spectrum was the [RCO+74]^+^ “monoacylglyceride” fragment at *m/z* 313 (Electronic Supplementary Material (ESM) Fig. S1).

**Fig. 3:**
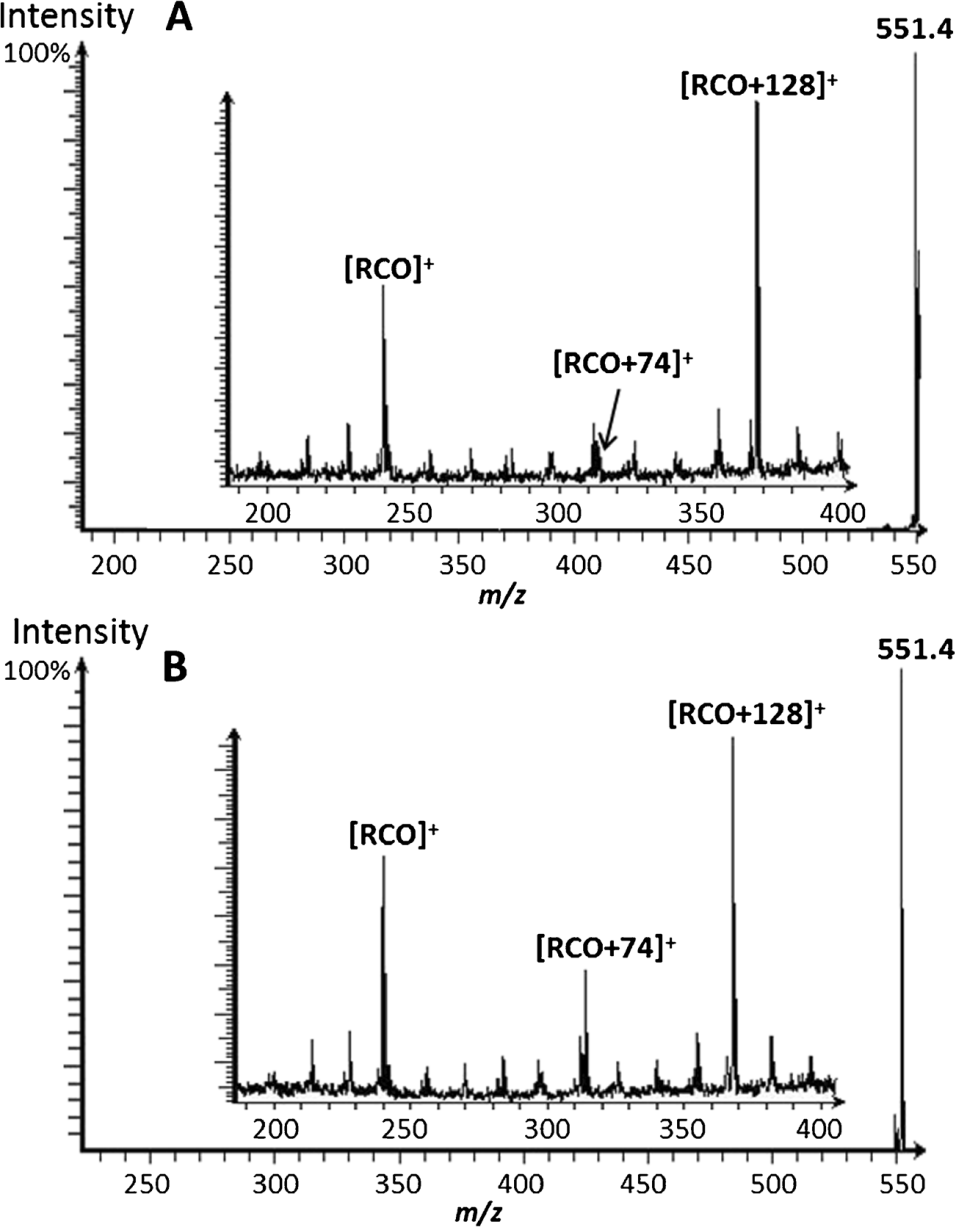
MS/MS spectra of the *m*/*z* 551.4 “DAG” peak in the spectra of (**A**) tripalmitate and (**B**) dipalmitate. The main characteristic productions detected are the [RCO]^+^ and [RCO+128]^+^ peaks. The [RCO+74]^+^ ion is approximately 50% of the intensity of the [RCO]^+^ peak in the dipalmitate spectrum but is barely visible in the tripalmitate sample

As expected, the MS/MS spectra of the *m/z* 551 ion produced many of the same product ions for both the di- and tripalmitate species (Fig. 3). The most intense fragment ion in both cases is the [RCO+128]^+^ peak at *m/z* 367 while the most significant difference is the absence of the [RCO+74]^+^ ion in the product ion spectrum generated from the tripalmitate. The gradual losses of hydrocarbons from the larger fragmented ions of DAG precursors can be observed in the MS/MS spectra as well.

The variation in the intensity of the [RCO+74]^+^ ion is extremely useful and suggests a possibility of distinguishing the origin of these isobaric ions in ToF-SIMS data.

**Fig. 4:**
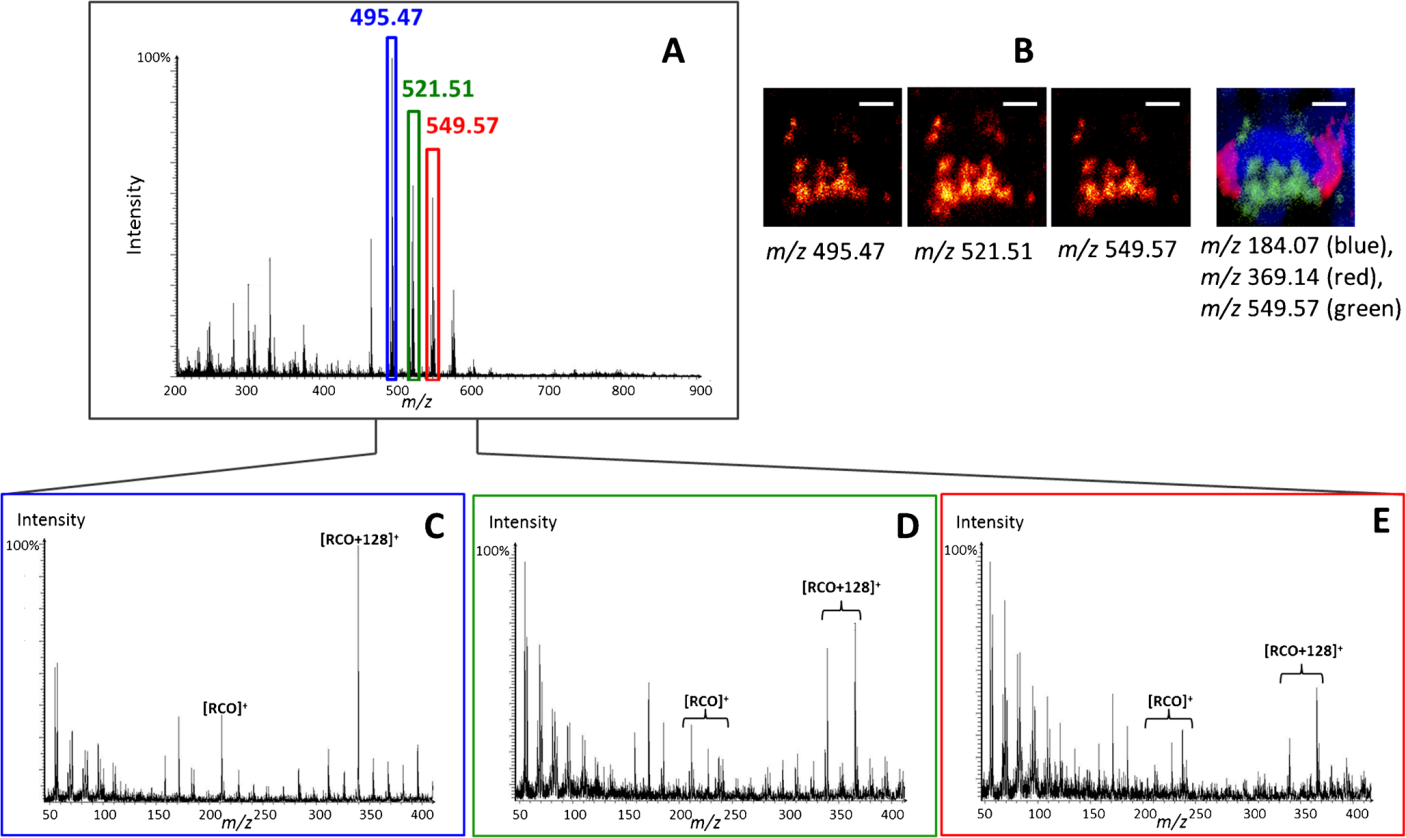
Structural elucidation of DAGs in the fly brain from SIMS MS/MS spectra. (**A**) MS spectrum of a fly brain with selected precursors *m/z* 495.47, 521.51, and 549.57 for further MS/MS. (**B**) SIMS MS ion images of the fly brain molecules. (**C–E**) SIMS MS/MS spectra of the selected precursors: (**C**) Potential DAG(28:0) ([M-OH]^+^, [C_31_H_59_O_4_]^+^) at *m/z* 495.5; (**D**) possible DAG(30:1) ([M-OH]^+^, [C_33_H_61_O_4_]^+^) at *m/z* 521.5; (**E**) possible DAG(32:1) ([M-OH]^+^, [C_35_H_65_O_4_]^+^) at *m/z* 549.6. *Scale bar* is 200 μm

For the data in Fig. 4, the selected precursors for possible DAG [M-OH]^+^ ions at *m/z* 495.47, 521.51, and 549.57 have been chosen (highlighted in Fig. 4A) and they distribute similarly in the proboscis of the fly head. In contrast, the PC headgroup fragment [C_5_H_15_PNO_4_]^+^ at *m/z* 184.07 distributes mainly across the central brain section, and the eye pigment at *m/z* 369.14 is localized in the eyes, next to the optical lobes (Fig. 4B). Detailed chemical distribution of lipids in the fly brain can be found in a previous publication [23]. The MS/MS data show various product ions observed for different parent ions; however, these product ions have similar patterns that are comparable with the di- and tripalmitate standards. From the observed product ions, one can calculate the carbon number and the unsaturation degree of the precursor compound. The peak at *m/z* 495.47 is indicative of DAG(28:0), and the MS/MS spectrum (Fig. 4C) contains intense product ion signals corresponding to [RCO]^+^ at *m/z* 221.2 and [RCO+128]^+^ ions at *m/z* 339.3 of a C14:0 fatty acid. The absence of an [RCO+74]^+^ peak in the product ion spectrum suggests that the origin of these species is TAG and not DAG.

For DAG(30:1) ([M-OH]^+^, [C_33_H_61_O_4_]^+^) at *m/z* 521.51, the peaks for [R_*n*_CO+128]^+^ ions at *m/z* 337.3, 339.3, 365.3, and 367.3 were detected indicating C14:1, C14:0, C16:1, and C16:0 fatty acid groups, respectively. The most intense of these four ions were those corresponding to the C14:0 and the C16:1. The associated [RCO]^+^ ions were also present along with [RCO+74]^+^ ions although they do not stand out above the hydrocarbon background making conclusive assignment to as DAG or TAG in this case.

In the case of DAG(32:1), the signal-to-noise ratio in the spectrum was not as high as in the other spectra, but there are still clear peaks assigned as [RCO+128]^+^ ions present at *m/z* 339.3, 365.3, and 367.3 along with a possible [RCO+128]^+^ peak at *m/z* 337.3. These species indicate the presence of C14:0, C16:1, C16:0, and possibly C14:1, respectively. The most abundant of these is the C16:1 species indicating that the precursor mainly contains C16:1 and C16:0. The presence of the C14:0-related ions suggests a second composition of C14:0 and C18:1 fatty acids. A peak corresponding to the [RCO+128]^+^ ion of the C18:1 fatty acid is present in the spectrum, but the intensity is not significantly higher than the hydrocarbon background. [RCO]^+^ peaks are observed accompanying the major [RCO+128]^+^ peaks, and no [RCO+74]^+^ ions are detected indicating TAG origins of these species.

### SIMS MS/MS imaging of the fly brain

**Fig. 5:**
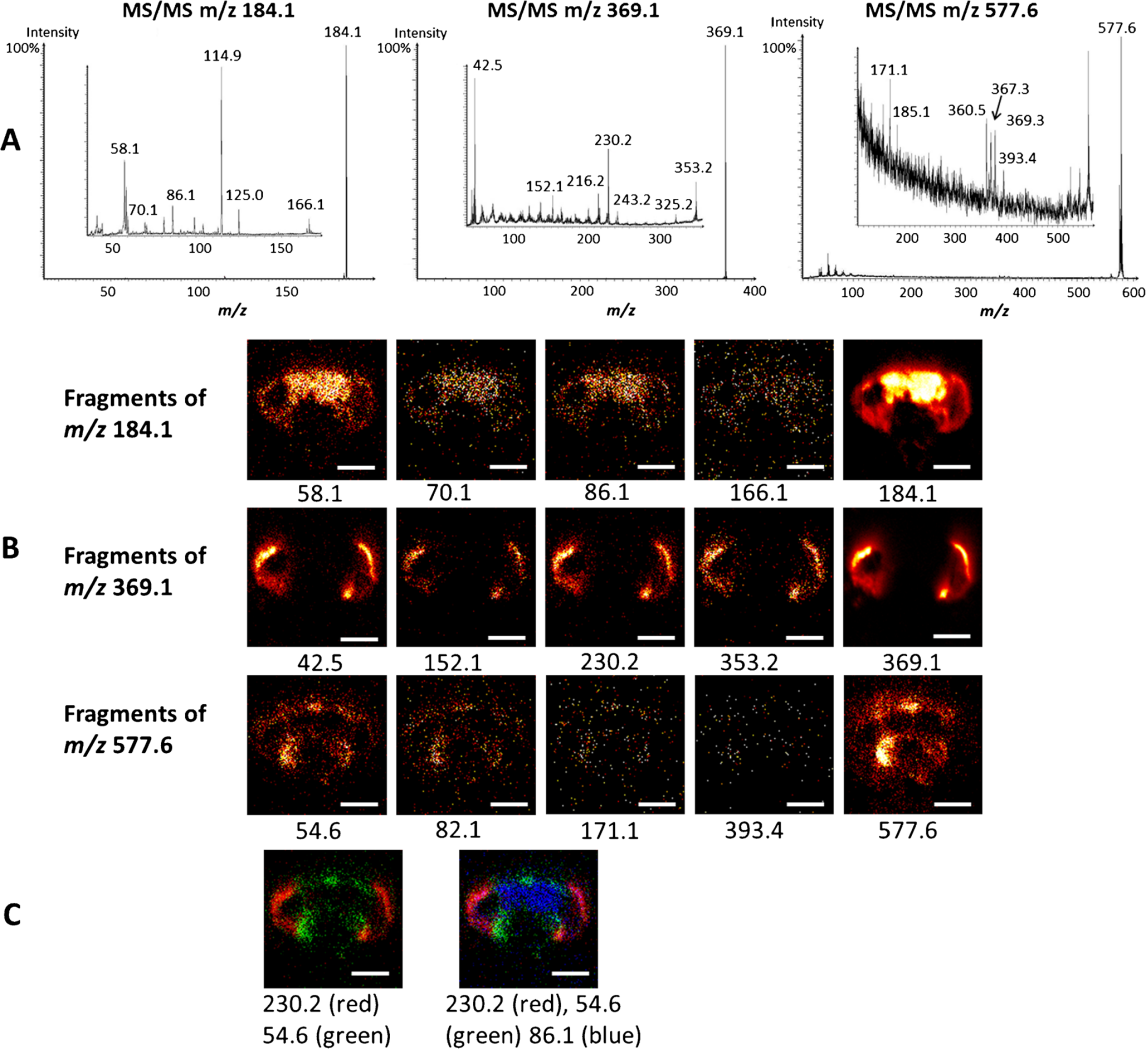
SIMS MS/MS imaging of fly brain. (**A**) MS/MS spectra of selected molecular precursors, PC headgroup *m/z* 184.1, eye pigment Drosopterins *m/z* 369.1, and (34:1) at *m/z* 577.6. (**B**) SIMS ion images of the corresponding MS/MS fragments of the molecular precursors. (**C**) Overlay of MS/MS ion images, 54.6 (*green*), *m/z* 230.2 (*red*), and 86.1 (*blue*). *Scale bar* is 200 μm. The *m/z* values are quoted to 0.05 for MS and 0.1 for MS/MS

In this section, we demonstrate the potential advantages of MS/MS imaging with SIMS. Combined with the structural identification of biomolecules, the distributions of their product ions can be investigated with MS/MS ion images. MS/MS spectra and images of the fly brain are shown in Fig. 5. The selected precursor ions are the PC headgroup at *m/z* 184.07, eye pigment drosopterins at *m/z* 369.14, and DAG(34:1) at *m/z* 577.57 from MS experiments. This produces MS/MS spectra with characteristic product ions. By examining the product ions at each pixel, we obtain their corresponding MS/MS images. The main fragments of PC headgroup, *m/z* 58.1, 70.1, [C_5_H_12_N]^+^ at *m/z* 86.1 and [C_5_H_13_NO_3_P]^+^ at *m/z* 166.1 distribute across the entire fly brain, but they are at relatively higher intensity in the central brain. The product ions of drosopterins such as *m/z* 152.1, 230.2, and 353.2 localize to the eyes next to the optical lobes as expected. Finally, the main product ions of DAG(34:1) for instance the fragments at *m/z* 54.6, 82.1, C_9_H_15_O_3_ at *m/z* 171.1, [RCO+128]^+^ ions, [C_25_H_45_O_3_]^+^ at *m/z* 393.4, and [C_23_H_43_O_3_]^+^ at *m/z* 367.3 localize around the proboscis and cuticle areas. Interestingly, the product ion spectra from the collision-induced decay (CID) of the *m/z* 577.6 ion also contain the peaks at *m/z* 360.5 and 369.3. These are attributed to interfering species at *m/z* 577.6 that in the case of the *m/z* 360.5 ion originates from the ITO glass substrate and in the case of the peak at *m/z* 369.3 is from the eye pigment. Retrospectively, reconstructing an MS/MS spectrum using only pixels from the proboscis and cuticle regions of the sample produces a result that does not contain these additional peaks (data not shown).

Most of the fragments have similar distributions to their precursors, however, with significantly lower signal intensities. The overlaid MS/MS ion images of representative fragments from each precursor are consistent with their overlaid MS ion images (Figs. 4B and 5C). The abnormally high peak at *m/z* 114.9 observed in the MS/MS spectrum of the PC headgroup is from an interference and will be discussed in the following section. The use of Ar_4000_^+^ GCIB in our study helped increase the signal intensities of molecular species to improve both MS/MS profiling and imaging.

### Deconvoluting ion distributions in the fly brain using MS/MS imaging

The ultimate purpose of SIMS MS/MS analysis is to identify the structure of the biomolecules of interest in the analysis. However, SIMS MS/MS imaging also offers an advantage in the ability to elucidate the distribution of a specific biomolecule based on the ion images of its fragmentation products. When interferences and ions of interest are present that have *m/z* too close to each other to be fully resolved by only MS, their MS ion images show the combined distribution of both species. This issue can be solved by tracking the product ion images of these species.

**Fig. 6:**
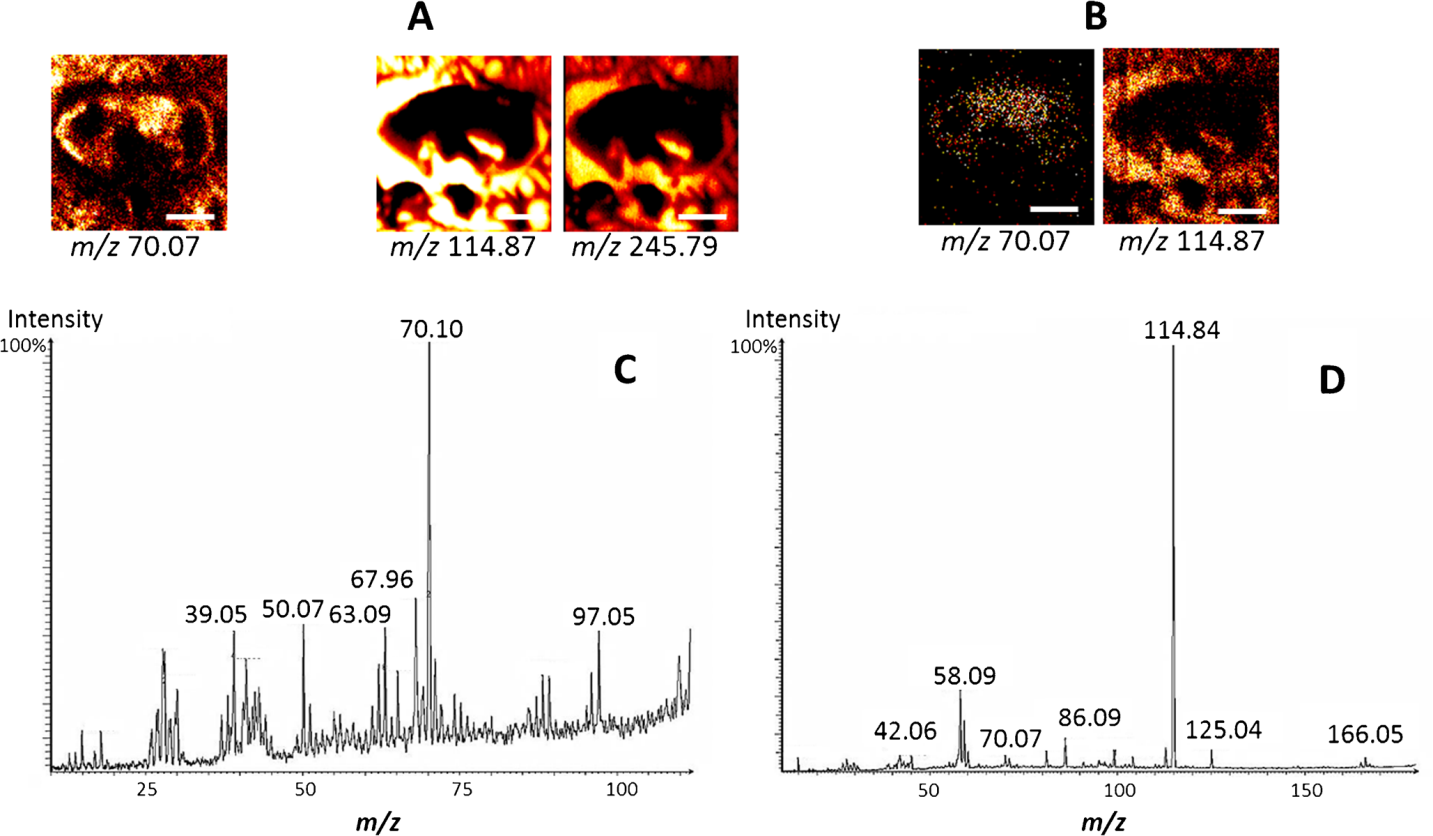
Elucidation of molecular distribution of the fly brain with MS/MS imaging. (**A**) SIMS ion images of MS ions at *m/z* 70.10, 114.87, and 245.79. (**B**) MS/MS spectrum of selected precursor *m/z* 114.9. (**C**) Ion image of MS/MS fragment at *m/z* 58.1, 70.1, and 114.9 from precursor PC headgroup *m/z* 184.1. (**D**) MS/MS spectrum of selected precursors *m/z* 184.1. The fragment *m/z* 70.1 is produced from both these precursors. The MS ion images of *m/z* 70.10 show the combined distribution of these fragments due to their very small difference in *m/z* (~0.03 Da). *Scale bar* is 200 μm. The *m/z* values are quoted to 0.05 for MS and 0.1 for MS/MS

In the fly brain, as in many other biological samples, there is a potential for interference owing to the very complex sample matrix. Sometimes isobaric interferences can be spotted by close inspection of peak shapes where peaks of similar, but not identical mass, may appear as shoulders on another peak. Generating accurate ion distributions of these overlapping peaks is very difficult. The application of MS/MS imaging to solve this is demonstrated in Fig. 6. MS ion images show that ions at *m/z* 70.10 distribute all over the fly brain and the area outside the brain (Fig. 6A). It is clearly difficult to know if this ion is actually one species or from different precursors by using only MS ion images. It is possible that one part is from a biomolecule within the brain and the other part is from interferences outside the brain having similar distribution to those of the substrate material, In^+^ at *m/z* 114.87, and In_2_O^+^ at *m/z* 245.79 (Fig. 6A). Further evaluation with MS/MS imaging of the PC headgroup at *m/z* 184.1, In^+^ at *m/z* 114.9, and [In_2_O]^+^ at *m/z* 245.8 (Fig. 6B–D) shows that the ion at *m/z* 70.1 could be a fragment of the PC headgroup or another species at *m/z* 184.1 and also from an unknown interference having similar mass to In at *m/z* 114.9. The fragment ion at *m/z* 70.1 was observed in the MS/MS spectrum of *m/z* 114.9, which is normally assumed to be In^+^ (Fig. 6B). On the other hand, the ion *m/z* 70.1 is also present in the MS/MS spectrum of the precursor *m/z* 184.1, and its localization is within the fly brain (Fig. 6C and D). This confirms that the ion at *m/z* 70.1 comes from both the *m/z* 114.9 and *m/z* 184.1 precursor species, and it distributes similarly to these precursors. This species is not identified as being fly brain specific in the normal MS image where the *m/z* 70.1 signal is observed both within and outside the tissue.

In addition, there was an abnormally high peak at *m/z* 114.9 observed in the MS/MS spectrum of precursor *m/z* 184.1 (Fig. 6D); however, its localization is outside the brain area which is clearly different from the PC head group. This suggests that there might be a small amount of background signal from the ITO glass falling at *m/z* 184 that would appear as chemical noise in the ion image of this species. Alternatively, it is also possible that the ion *m/z* 70.1 detected outside the brain is actually a MS^3^ fragment of the ion at *m/z* 114.9 which is a MS^2^ fragment of an interference having *m/z* closed to *m/z* 184.1. The interference could be from the surrounding gelatin that was used to embed the fly head. Thus, the fragments of the interference are observed in the MS/MS spectra of the PC headgroup *m/z* 184.1 and In^+^. It is clear in this situation, with MS/MS analysis and imaging, that it is possible to distinguish and interpret the interference signal to obtain biologically meaningful information. This example demonstrates both the complexity associated with interpreting complex ToF-SIMS data from biological samples and the potential power of imaging MS/MS for helping decode this complexity.

## Conclusions

We demonstrate MS and MS/MS profiling and imaging and its application on the J105 SIMS instrument using the 40-keV Ar_4000_^+^ GCIB to study the lipid structure of *Drosophila* brain. MS/MS profiling is used to confirm the molecular structure of biomolecules from low mass ion species to intact molecular lipids. For glycerophospholipids, the types of lipids and fatty acid components can be identified based on their headgroup fragments and fatty acid product ions and for the first time in SIMS analysis a possible means of establishing the origin of the so-called DAG peaks is presented. Furthermore, MS/MS imaging offers a unique possibility for detailed elucidation of biomolecular distribution with high accuracy. This is particularly useful in the presence of the interferences which disturb the interpretation of biomolecular localization. The MS/MS imaging approach would be also very useful in biological applications studying the relation between biomolecules and their synthetic precursors or metabolic products, for instance, the involvement of a fatty acid in the synthesis or metabolism of lipids.

## Electronic supplementary material

Fig. S1 (PDF 262 kb)
