# Supplementary material for: MS/MS analysis and imaging of lipids across Drosophila brain using secondary ion mass spectrometry
Source: Anal Bioanal Chem. 2017 Apr 7;409(16):3923–32. doi: 10.1007/s00216-017-0336-4 (PMC5437193; doi:10.1007/s00216-017-0336-4)
Supplement: Supplementary file 1 — (PDF 262 kb) [file 216_2017_336_MOESM1_ESM.pdf]

Analytical and Bioanalytical Chemistry

Electronic Supplementary Material

MS/MS analysis and imaging of lipids across *Drosophila* brain using secondary ion mass spectrometry

Nhu T. N. Phan, Marwa Munem, Andrew G. Ewing, John S. Fletcher

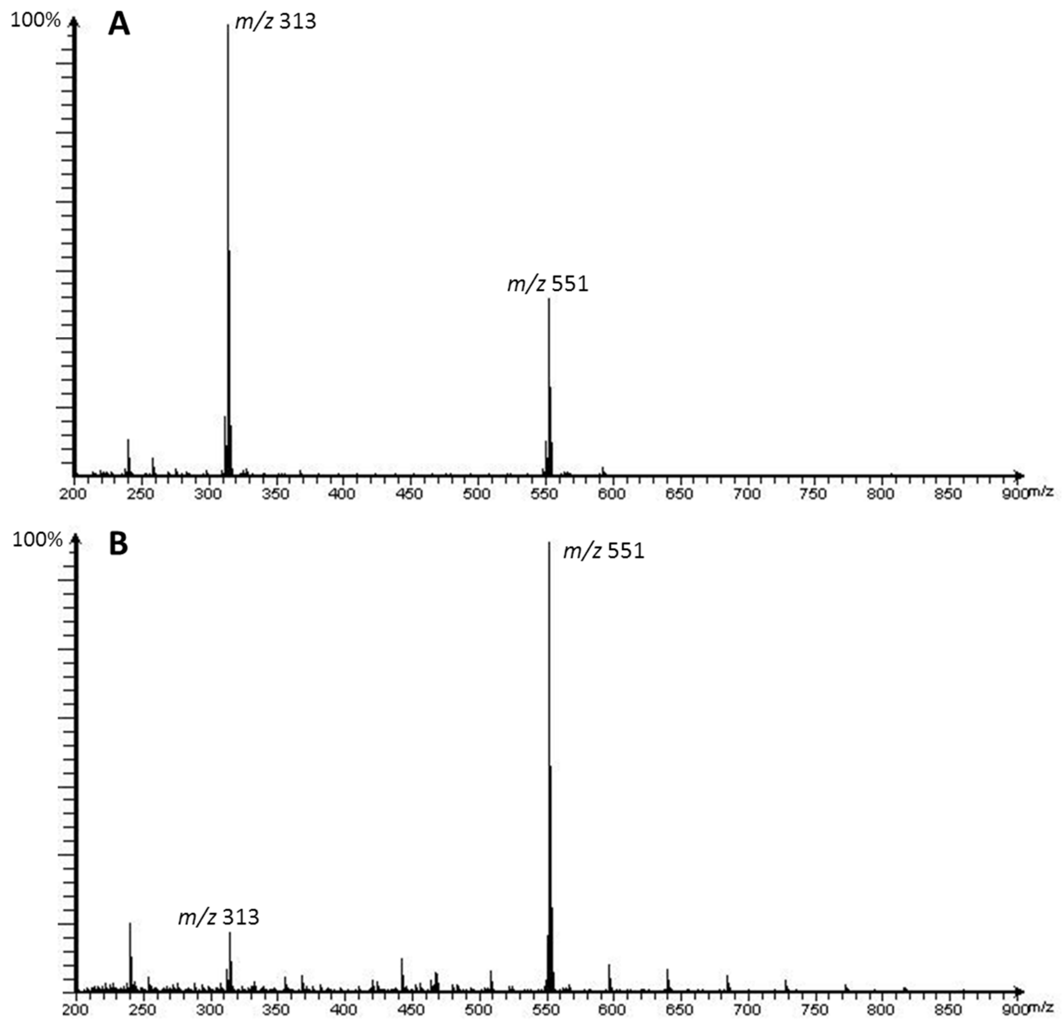

Fig. S1 Reference ToF-SIMS spectra of dipalmitate (a) and tripalmitate (b). The both spectra show the $[RCO+74]^+$ ion at *m/z* 313 and a peak at *m/z* 551 assigned as $[M\text{-}OH]^+$ for the dipalmitate and $[M\text{-}RCOO]^+$ for the tripalmitate
